# Cut the fat: targeting cholesterol and lipid metabolism in glioblastoma

**DOI:** 10.1038/s41419-025-07993-3

**Published:** 2025-10-07

**Authors:** Safet Zekanovic, Puspha Achaiber Sing, Sieger Leenstra, Martine L. M. Lamfers

**Affiliations:** Department of Neurosurgery, Brain Tumor Center, Erasmus MC Cancer Institute, Erasmus University Medical Center, Rotterdam, Netherlands

**Keywords:** Target identification, Cancer metabolism, Lipids

## Abstract

In the past decades, much research has been focused on identifying oncogenic signaling events in glioblastoma (GBM). Based on these findings, novel therapeutics have been extensively tested in clinical trials. These include targeted agents (e.g., kinase inhibitors), anti-angiogenic drugs, and immunotherapies. Unfortunately, no survival benefit has been demonstrated with the use of these agents. The lack of efficacy observed in the past decades poses the question of whether we are targeting the right pathways to halt the growth of GBM. In this review article, we discuss the intricacies of cholesterol and lipid metabolism in GBM. We provide an overview of which oncogenic signaling pathways are fueled by the aberrant cholesterol and lipid metabolism in GBM. Importantly, we also discuss how metabolic rewiring in the context of cholesterol and lipids can contribute to immune evasion in GBM. Lastly, we provide an overview of current drugs targeting cholesterol and fatty acid metabolism and their potential to serve as targeted therapies for GBM.

## Facts

- Glioblastoma is a CNS malignancy in dire need of novel (targeted) therapies.
- Cholesterol and lipid metabolism rewiring can be up- or downstream of established oncogenic signaling pathways.
- Rewiring of cholesterol and lipid metabolism contributes to tumor immune evasion through T-cell dysfunction and myeloid cell polarization.
- Non-oncological drugs targeting cholesterol and lipid metabolism have antitumoral properties.

## Open questions

- Can lipid raft integrity be selectively targeted in GBM cells?
- Can induction of cell differentiation attenuate the metabolic plasticity of GBM cells?
- Is there any clinical effect of current drugs targeting cholesterol and lipid metabolism?
- Does targeting cholesterol and lipid metabolism hold promise in augmenting the efficacy of immunotherapies?
- Which targets should be pursued in the future to tackle the tumor-promoting effects of metabolic rewiring in GBM?

## Introduction

Glioblastoma isocitrate dehydrogenase 1 wild type (IDHwt GBM, hereafter GBM) remains one of the most aggressive forms of cancer, accounting for approximately 48% of all malignant brain tumors [1]. Previously known as glioblastoma multiforme, histological analysis reveals an invasive growth pattern with areas of necrosis, perivascular cuffing, neovascularization, high numbers of myeloid infiltrates, and the presence of lipid droplets [2–4]. Furthermore, glioblastoma is characterized by its high inter- and intratumoral heterogeneity, greatly complicating treatment [5]. Current treatment options consist of surgical resection followed by radiation and chemotherapy with the alkylating agent temozolomide (TMZ) [6]. The angiogenesis inhibitor bevacizumab gained approval in the US for the treatment of recurrent GBM, although its efficacy is limited in terms of prolonging survival [7]. In the past two decades, no new treatment options have been approved for GBM, despite extensive research efforts and numerous clinical trials [8–10]. A wide range of agents targeting diverse pathways has been investigated in (pre)clinical studies, yet these trials have yielded limited success [11–13]. This underscores the need for alternative approaches, as some aspects of GBM tumor biology remain underexplored and could serve as a starting point for the identification of new therapeutic targets. One of these aspects is the dysregulated cholesterol and lipid metabolism in GBM and how it influences GBM proliferation, invasiveness, sensitivity to radiochemotherapy, and immune evasion. In this review, we highlight findings on aberrant cholesterol and lipid metabolism in GBM and how these phenomena contribute to its growth, immune evasion, and therapeutic resistance. We also briefly discuss the antitumor effects of non-oncological drugs that target cholesterol or lipid metabolism and their potential to combat GBM. Improved understanding of GBM tumor biology will provide new insights in terms of target discovery and aid in drug development for GBM.

## Glioblastoma biological behavior: what do we know now?

### Glioblastoma subtypes reflect their plasticity and heterogeneity

Genetic, epigenetic, and microenvironmental factors shape cellular processes and contribute to the diversity of GBM. In vivo modeling of early gliomagenesis revealed that cerebral precursor cells acquire a neural crest-like signature and that this signature is transiently present during tumor formation [14]. Glial progenitors, however, are deemed the major contributing factor to GBM heterogeneity as these give rise to several cell types, some of which only transiently present in gliomagenesis [15]. Further efforts in unraveling this heterogeneity have resulted in the classification of GBM into four transcriptional subtypes: mesenchymal-like (MES-like), astrocyte-like (AC-like), oligodendrocyte-progenitor-like (OPC-like), and the neural-progenitor-like (NPC-like) state [16]. Neftel el., demonstrated that all GBM tumors display characteristics of at least two of these subtypes, and many tumors harbor genetic alterations belonging to all four cell states [17]. AC-like tumors are associated with amplifications of the gene encoding the epidermal growth factor receptor (EGFR) and annexin A1 (ANXA1), the latter potentially involved in the polarization of macrophages to the tumor-supporting M2 phenotype [18]. NPC-like tumors often harbor cyclin-dependent kinase 4 (CDK4) amplifications and are possibly more prone to T-cell mediated cytotoxicity conferred by a higher expression of chemerin (RARRES2) [19]. Loss of neurofibromin 1 (NF1) function and cellular communication network factor 1 (CCN1) upregulation are characteristic of MES-like GBM and represent defects in the negative regulation of the Ras-pathway and increased recruitment of tumor-supporting macrophages, respectively [20]. OPC-like tumors are associated with amplifications of platelet-derived growth factor receptor alpha (PDGFRA) and increased expression of brain-expressed X-linked 1 and 4 (BEX1/BEX4), the latter promoting invasion and migration of GBM cells [21].

### Hyperactivity of the EGFR/PI3K/Akt/mTOR pathway contributes to GBM growth

The EGFR/PI3K/Akt/PTEN/mTOR pathway has been shown to be critical in glioma cell growth and proliferation [22]. Amplification of the gene encoding the epidermal growth factor receptor and in-frame deletion of exons 2-7, resulting in the truncated EGFR variant III (EGFRvIII), has been established as a key driver in GBM. EGFR is overexpressed in approximately 50% of GBM patients, with the vIII variant present in about half of those with EGFR amplification [23–25]. EGFR promotes oncogenic signaling events via its phosphorylating potential, ultimately leading to downstream effects like proliferation, cytokine release, and cell cycle progression [26, 27]. The presence of EGFRvIII confers a constitutively active receptor that facilitates cell growth and proliferation, but also enables glioma cells to evade apoptosis through binding to p53-upregulated modulator of apoptosis (PUMA), thereby preventing apoptotic signaling cascades [28]. The phosphoinositide-3-kinase (PI3K) protein can be activated by a myriad of upstream regulators, including EGFR, and mediates tumorigenesis, cellular proliferation, apoptosis, and cell invasion [29, 30]. PI3K, when activated, generates phosphatidylinositol [3–5]-trisphosphate (PIP3), which is necessary for the phosphorylation of Akt (a serine/threonine kinase). Akt in turn activates the mammalian target of rapamycin (mTOR,) leading to oncogenic signal transduction resulting in growth, lipid synthesis, and proliferation [31]. Finally, the mammalian target of rapamycine (mTOR) might be one of the bridges between GBM cell metabolism and epigenetic plasticity, as demonstrated by studies showing mTOR is able to modulate histone methylation and thereby contribute to tumor growth [32–34].

### Inactivation of tumor suppressors

GBM is well known to thrive due to the inactivation of tumor suppressor genes (TSGs) leading to activation of oncogenes, uncontrolled cell division, and resistance to apoptotic stimuli [35–37]. One of the most important TSGs that is frequently mutated in GBM is p53. In GBM, p53 mutations mostly lead to loss-of-function, gain-of-function, and dominant-negative effects [38]. Considering the pivotal role of p53 in tumor suppression, many oncogenic signaling pathways are able to exert their protumoral effects due to a defective p53 pathway. Retinoblastoma 1 (Rb) is another TSG that is frequently inactivated in GBM. Rb is known to function as a negative regulator of cell cycle progression by modulating the transcriptional activity of E2F transcription factor 1 (E2F1), hereby inhibiting transcriptional targets necessary for the S-phase of the cell cycle [39, 40]. Another key player in tumor suppression is the phosphatase and tensin homolog (PTEN). PTEN is involved in the negative regulation of the Akt pathway and prevents oncogenic signaling by dephosphorylating Akt, ultimately preventing it from activating mTOR, where activation of the latter promotes tumor cell survival, cell cycle progression, and enhanced protein synthesis [41–43]. Lastly, mutations in neurofibromin 1 (NF1), a negative regulator of the Ras protein, are observed in 10-15% of GBM patients [44]. Loss of NF1-mediated negative regulation of Ras leads to enhanced GBM cell growth and proliferation through the RAF-MEK-ERK signaling cascade [45]. Taken together, inactivation of tumor suppressors in GBM contributes greatly to its uncontrolled cell growth and proliferation.

### Role of glioblastoma stem-like cells

Glioblastoma is known for its heterogeneity, consisting of various subpopulations that contribute to proliferation, neovascularization, invasion, and radio-and chemoresistance [46–48]. To enable GBM cells to display such a high degree of plasticity, maintenance of stem cell characteristics is indispensable. One of the markers often identified in GBM contributing to its stem-like properties is the SRY-box transcription factor 2 (SOX2) [49]. SOX2 has been shown to be essential for GBM cell propagation by inhibiting the activation of tumor suppressors [50]. SOX2-positive GBM cells also show a higher potential for invasion relative to their SOX2-negative counterparts, emphasizing the pleiotropic effects of SOX2 [51, 52]. Another marker strongly associated with tumor cell stemness is the hyaluronan receptor CD44 [53]. CD44 expression is shown to contribute to cell migration and invasion, factors that pose major challenges in tackling GBM [54]. By contrast, a biphasic relationship of CD44 is postulated by Klank et al., where intermediate CD44 levels are associated with increased GBM cell migration but high levels with better patient outcomes [55].

CD133 (prominin-1) has been identified as another pivotal marker of GBM stem-like behavior. Its tumor-promoting effect is characterized by its ability to activate the p85 regulatory subunit of the PI3K protein, thereby leading to recruitment of Akt to the plasma membrane. This process induces the activation of the PI3K/Akt pathway, ultimately leading to GBM cell self-renewal and tumor formation [56]. CD133^+^ cells also display a high level of radiochemoresistance, suggesting that CD133 has pleiotropic effects in GBM cells [57, 58]. Bao et al. demonstrated a higher efficiency of DNA repair in CD133^+^ GBM cells after irradiation through a stronger upregulation of DNA checkpoint proteins relative to CD133-negative GBM cells [59]. Moreover, Nakai et al. found an increased expression of the multidrug resistance transporter 1 (MDR1) in CD133^+^ GBM cells, indicating that CD133 might promote drug efflux as well [60].

### GBM tumor microenvironment

GBM is strongly influenced by its tumor microenvironment (TME). The TME is characterized by the infiltration of many cell types, mostly tumor-associated macrophages (TAM), tumor-associated astrocytes (TAA), vascular endothelial cells, and neurons, all contributing to glioblastoma growth and proliferation through distinctive processes. Although generally considered as an immunologically ‘cold’ tumor, GBM can be classified into three subtypes, TME^HIGH,^ TME^MED^, and TME^LOW^, depending on the extent of myeloid and lymphoid cell infiltration. Each subgroup also displays a differential expression pattern of immune checkpoint molecules (e.g., PD-1 and CTLA-4) and is therefore less or more prone to antitumor immune responses elicited by immune checkpoint therapy, with TME^HIGH^ tumors being more prone [61]. Neurons have been demonstrated to promote GBM growth through the formation of the neurogliomal synapse. Here, AMPA receptors play a crucial role in promoting excitatory activity by enhanced glutamatergic inputs at the neurogliomal synapses [62]. The GBM-astrocyte crosstalk will be discussed later in this review, as the involvement of astrocytes is characterized by a more intricate communication between the two cell types.

## Switching gears: cholesterol and lipid metabolism in GBM

### Failure of targeted drugs in GBM: are we looking at all the right targets?

Over the past two decades, clinical trials in GBM have commonly evaluated the therapeutic activity of targeted drugs with established efficacy in other cancers. Unfortunately, none of these clinical trials revealed a survival benefit, even for brain-penetrant compounds such as the PI3K-inhibitor buparlisib and the PARP inhibitor veliparib [9, 63–65]. In addition, the highly anticipated antibody-drug conjugate depatuxizumab mafodotin (depatux-m) targeting EGFR(vIII) also failed to extend survival in a phase III trial in GBM patients [66]. This raises the question of whether the most critical intracellular events in GBM are being targeted. Emerging research on metabolic rewiring in GBM has revealed its role in promoting proliferation, angiogenesis, immune evasion, and radiochemoresistance. Preclinical studies targeting metabolic (hyper)activity (mainly modulated by the Warburg effect) appeared promising for the treatment of GBM [67–69]. However, none of these approaches has led to treatment efficacy in clinical trials [70–72]. Focusing on other key players in the aberrant metabolism in GBM may shed new light on GBM biological behavior and create alternative opportunities for target finding and drug development. In this context, cholesterol and lipid metabolism in GBM remain relatively unexplored.

### Physiology of cholesterol and lipid metabolism in the CNS

Cholesterol is an essential organic molecule that has a variety of functions in the human body. Its main functions are the maintenance of cell membrane fluidity and permeability, insulating nerve fibers in the central nervous system, synthesis of steroid hormones/vitamins, and acting as a cofactor for many signaling molecules [73]. The brain contains nearly a quarter of all cholesterol in the body, as it is a major component of myelinated membranes in white matter in the brain [74, 75]. The synthesis of cholesterol is a multistage process for which extensive literature is available [76]. In short, cholesterol biosynthesis requires insulin, NADPH, and other enzymes, including the most studied one, 3-hydroxy-3-methylglutaryl-CoA reductase (HMGCR) [77]. Cholesterol homeostasis in the brain is unique relative to other parts of the human body due to the fact that cholesterol is not able to cross the blood-brain barrier (BBB), meaning this steroid must be synthesized locally [78]. Exchange of cholesterol between cells in the human brain is mediated by low-density lipoprotein receptors (LDLR), which control the uptake of cholesterol in the form of lipoproteins [79]. Another important mediator of cholesterol homeostasis is the sterol regulatory element binding transcription factor (SREBP1). SREBP1 functions as an intracellular sensor that, under low levels of cholesterol metabolites, translocates to the nucleus to activate genes involved in cholesterol and lipid biosynthesis [80, 81].

### GBM displays a rewired cholesterol metabolism

How GBM cells obtain their cholesterol differs significantly from other cell types in the CNS. A study by Villa et al. demonstrated that GBM cells do not employ de novo cholesterol synthesis as astrocytes do. Instead, GBM cells shut down proteins necessary for de novo cholesterol synthesis and rely on the uptake of extracellular cholesterol [82]. This shutdown is necessary to prevent the intracellular machinery, responsible for cholesterol homeostasis, from activating ATP-binding cassette subfamily A member 1 (ABCA1). The latter serves as an efflux transporter to prevent intracellular cholesterol accumulation [83]. Other studies, also showing the independence of GBM cells on de novo cholesterol biosynthesis, demonstrate that GBM cells take up cholesterol through LDLR, which is upregulated by the EGFR/PI3K/Akt pathway [84, 85]. Moreover, a study by Yuan et al. found that a higher LDLR expression is associated with a stronger expression of immune checkpoint molecules CTLA4 and LAG3 and an immunosuppressive TME, indicating that LDLR might promote tumor growth in multiple ways [86]. The notion that GBM cells steer away from de novo cholesterol synthesis and rely on exogenous cholesterol warrants further investigation, as this may present an important lead for novel targeted therapy approaches.

### Intermediates in cholesterol biosynthesis can exert protumoral effects

Intermediates for cholesterol biosynthesis can also promote GBM growth and proliferation. Considering the fact that cholesterol biosynthesis consists of approximately 30 steps, highlighting every intermediate’s potential tumor-promoting effect is outside the scope of this review [87]. However, we will describe some intermediates for which there is evidence of their protumoral effects. One of the first intermediates, mevalonate, has been demonstrated to be downstream of MYC, and inhibition of mevalonate synthesis with simvastatin (an inhibitor of HMGCR) was demonstrated to prolong the survival of brain tumor-bearing mice [88]. Furthermore, expression of farnesyl diphosphate synthase (FDPS) has been demonstrated to be higher in GBM patient samples relative to normal human astrocytes (NHA) and peripheral tumor-free brain tissue. FDPS levels were correlated to phosphorylation levels of Akt and ERK, thereby contributing to oncogenic signaling [89]. Kim et al. demonstrated an antitumor effect of FDPS inhibition, which attenuated an embryonic stem cell phenotype and promoted apoptosis induction in GBM cells [90]. Strengthening the putative protumoral role of FDPS in GBM is the observation that paclitaxel’s cytotoxic effects were inhibited by FDPS through Ras activation [91]. Another enzyme in the biosynthesis of cholesterol, farnesyl-diphosphate farnesyltransferase 1 (FDFT1), promotes GBM cell invasion and migration, possibly through upregulation of p-Akt [92]. Collectively, these observations emphasize the involvement of not only cholesterol but also its precursors in GBM growth and proliferation.

### Products of cholesterol metabolism can contribute to GBM progression

Cholesterol itself is prone to a variety of metabolic conversions that each serve their purpose in steroid hormone production, vitamin D synthesis, and intracellular signaling [93–95]. Interestingly, products of cholesterol metabolism exert different effects that are influenced by their concentration, tissue expression, and signaling context. Geng et al. demonstrated that patient-derived GBM tissue contains high concentrations of cholesterol esters and that lipophagy promotes liberation of these esters from lipid droplets to increase intracellular cholesterol levels [96]. By contrast, high levels of 24-hydroxycholesterol have cytotoxic effects on GBM cells by acting as a ligand for the liver-X-receptor (LXR), an important intracellular sensor for cholesterol metabolism. Activation of LXR by 24-hydroxycholesterol led to an increase in caspase 3/7 and subsequent induction of apoptosis [97]. Bhat et al. revealed an extended survival of mice treated with a dopamine receptor antagonist and irradiation, which was accompanied by an increase in cholesterol esters. This implies that cholesterol esters might exert antitumoral effects as well, dependent on the specific ester of cholesterol [98]. The above highlights the versatility of cholesterol metabolites in their functions in tumor cell growth, proliferation, and cell death.

### Cholesterol-containing lipid rafts are indispensable for tumor cells

**Fig. 1:**
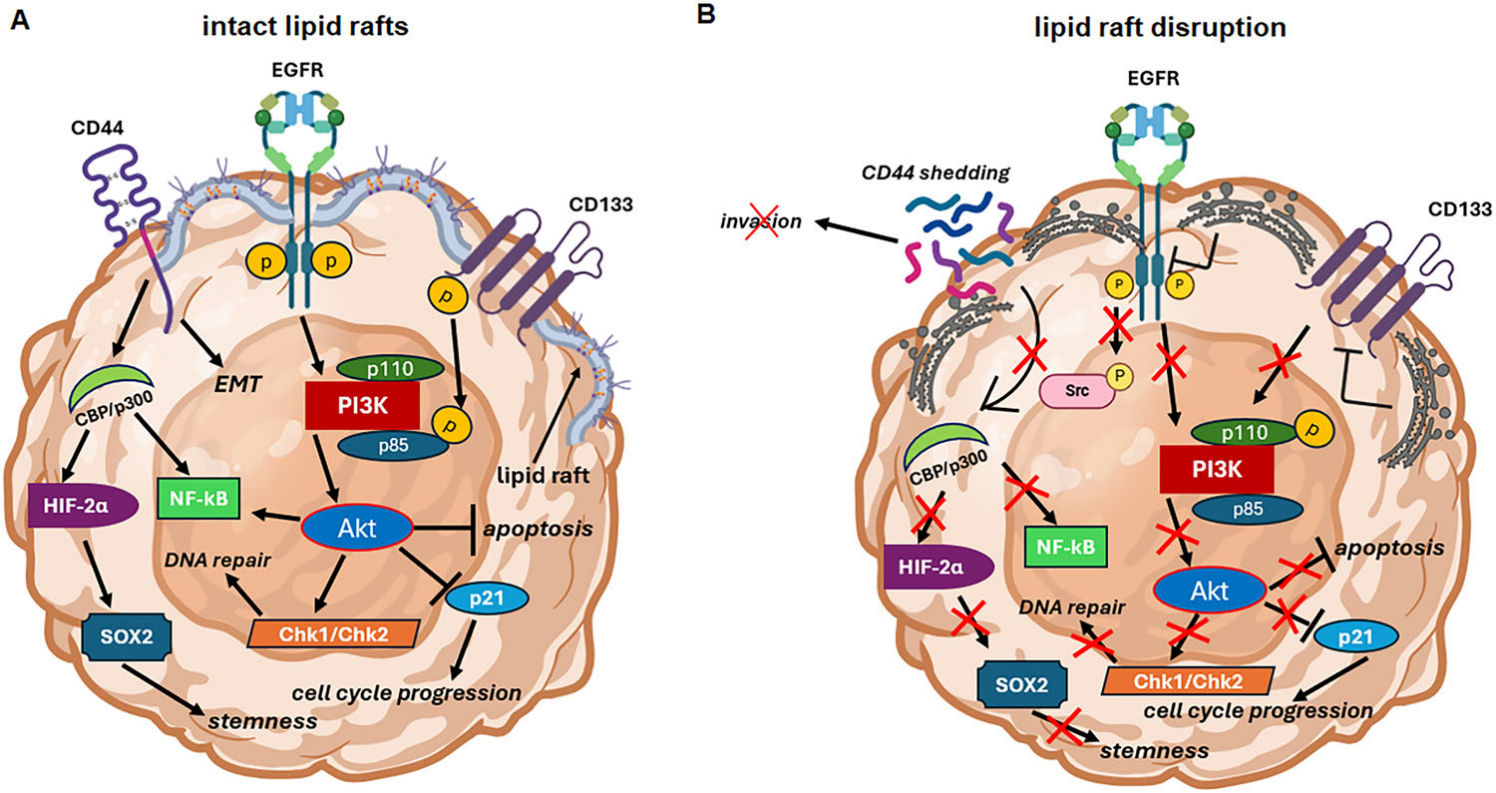
Cholesterol is an essential component of lipid rafts and ensures the correct localization of transmembrane proteins. **A** Lipid rafts are crucial in the maintenance of signaling platforms in tumor cells by supporting the proper docking of various oncogenic receptors and stemness proteins. **B** disruption of lipid raft integrity has deleterious effects on oncogenic signaling pathways like EGFR/PI3K/Akt. Lipid raft disruption affects transmembrane stemness proteins (e.g., CD44 and CD133) as well, hereby attenuating the plasticity and self-renewal capacity of tumor cells. Chk1/Chk2 checkpoint kinase 1/2 EMT epithelial-to-mesenchymal transition, CBP/p300 CREB-binding protein/E1A binding protein p300, HIF-2α hypoxia inducible factor 2 alpha, EGFR epidermal growth factor receptor, NF-kB nuclear factor kappa B, p21 cyclin-dependent kinase inhibitor 1A PI3K phosphoinositide-3-kinase, CD133 prominin-1, CD44 hyaluronate receptor, Akt protein kinase B, SOX2 SRY-box transcription factor 2. Figure created with BioRender.com.

An emerging role for cholesterol itself in oncogenic signaling events has been proposed for a plethora of other cancers [99–101]. Dos Santos et al. found that LDL-cholesterol accelerated breast cancer growth in hypercholesterolemic diet-fed mice. This promotion of cancer cell growth was demonstrated to be a consequence of mitogen-activated protein kinase 1 (MAPK1) and Erb-B2 receptor tyrosine kinase 2 (ERBB2) upregulation [102]. Importantly, cholesterol seems to be indispensable for the maintenance of lipid rafts in the cell membrane [103]. A study conducted by Lasserre et al. demonstrated that lipid nanodomains are essential for activation of the PI3K/Akt pathway by inducing the recruitment of Akt to the cell membrane, hereby facilitating oncogenic signaling events [104]. Not only does the PI3K/Akt pathway seem to be modulated by lipid rafts. Transmembrane proteins like oncogenic receptors (EGFR/EGFRvIII) and stemness markers (e.g., CD44 and CD133) also require proper docking in the cell membrane [105, 106]. In addition, NF-kB has been identified as one of the key regulators of a pro-inflammatory tumor-promoting environment in GBM [107]. Song et al. observed a decrease in expression of NF-kB when one of the main components of lipid rafts, flotilin-2 (FLOT2), was depleted, further strengthening the oncogenic role of lipid rafts in cancer [108]. Targeting lipid rafts might attenuate oncogenic signaling events in GBM and therefore offer an interesting target to combat GBM (Fig. 1).

### GBM cells require cholesterol to maintain their stemness

GBM cells display a high degree of stemness, enabling them to respond and adapt quickly to environmental cues, insult, and (oxidative) stress. This stemness is also associated with inherent and acquired therapeutic resistance. How cholesterol metabolism in GBM cells contributes to stemness remained elusive for a long time. Transmembrane stemness proteins require proper functioning lipid rafts to exert their oncogenic function. CD133 (prominin-1) is localized in lipid rafts as a transmembrane protein [109]. Its function has been demonstrated to be highly dependent on intact lipid rafts, where its intracellular portion is able to stimulate PI3K (see “Intermediates in cholesterol biosynthesis can exert protumoral effects“) [110]. CD44 is a marker that has been implicated to confer a worse prognosis in low-grade GBM patients and has been demonstrated to be involved in the maintenance of protumoral M2 macrophages and GBM invasion [111–113]. Cholesterol depletion and subsequent disruption of lipid rafts led to shedding of CD44 and diminished GBM cell migration and invasion [114, 115]. A study by Marks et al. found that expression of HMGCR promotes stemness in a breast cancer model as observed by an upregulation of SOX2, CD44, and CD133 [116]. Taken together, cholesterol-containing lipid rafts and HMGCR expression can promote the stem-like behavior of GBM cells.

## The tumor microenvironment – GBM crosstalk involves cholesterol and lipids

### Astrocytes: more than just spectators

In the past, tumor-associated astrocytes (TAA) were considered mere bystanders in GBM. This view has changed considerably over the past few years after the realization that understanding GBM biology requires a holistic approach. Heiland et al. demonstrated that astrocytes residing at the tumor margin are able to promote an anti-inflammatory environment to support immune evasion of GBM cells through the release of TGF-β and IL-19 [117]. Additionally, secretome profiling revealed that astrocytes, when co-cultured with CD133^+^ GBM cells, secrete pro-invasive chemokines like TGF-β and CXCL12, aiding in GBM cell invasion [118]. GBM cells are potentially able to shut down de novo cholesterol synthesis and rely on exogenous uptake thereof (see “Metabolic rewiring in microglia promotes GBM immune evasion“). Astrocytes appear to play a role in this switch by enhancing their de novo cholesterol synthesis, possibly due to amyloid-β and glutamate exposure mediated by GBM [119–121]. Moreover, Perelroizen et al. revealed that cholesterol secreted by astrocytes is indispensable for GBM survival as depletion of astrocytes led to detrimental metabolic changes in GBM cells [122]. The emerging role of astrocytes in GBM growth emphasizes the importance of considering intercellular crosstalk within the TME for the development of novel treatments.

### Cholesterol and lipids: immune evasion tactics through CD8 + T-cell impairment

Cholesterol metabolism not only mediates oncogenic signaling events, stemness and astrocyte cross-talk, but might also be directly involved in immune evasion. In a melanoma model, Ma et al. found that an increased cholesterol content in the TME leads to cholesterol accumulation in CD8 **+** T-cells [123]. This cholesterol accumulation renders CD8 **+** T-cells exhausted and is accompanied by an increased expression of immune checkpoint molecules like PD-1 and 2B4. Apoptotic activity was also higher in CD8^+^ T-cells enriched in cholesterol. Administration of simvastatin (an inhibitor of cholesterol synthesis) in lung tumor-bearing mice reduced the cholesterol content in the tumor and its TME, subsequently leading to PD-1 and 2B4 downregulation. Further substantiating the immune evasive effects of cholesterol is the observation that 27-hydroxycholesterol (27-HC) is able to impair CD8 **+** T-cell function through apoptosis induction mediated by macrophages that are treated with 27-HC [124]. In addition, cholesterol metabolites (e.g., 22-HC and 25-HC) released by tumor cells can inhibit CCR7 expression on maturing dendritic cells, thereby leading to impairments in antigen presentation and development of immunological tolerance in naive T-cells, further contributing to immune evasion [125].

### Metabolic rewiring in microglia promotes GBM immune evasion

Cholesterol metabolism rewiring affects not only T-cell function and activity but also myeloid cell behavior. Microglia in recurrent GBM demonstrate a higher expression of genes involved in fatty acid oxidation as demonstrated by upregulation of phospholipase D family member 3 (PLD3) and pyruvate dehydrogenase kinase (PDK4), among others [126]. PLD3 promotes cleavage of single-stranded DNA (ssDNA, potentially derived from necrotic GBM cells), which might impair toll-like receptor 9 (TLR9) activation and hamper the development of pro-inflammatory antitumoral macrophage responses [127–129]. Thus, PLD3-mediated attenuation of TLR9 signaling could possibly inhibit the polarization of M2 microglia to the antitumoral M1 subset. Interestingly, microglia subjected to oxidative stress rewire their cholesterol metabolism through increased squalene epoxidase (SQLE) expression, leading to decreased antigen presentation to CD8 **+** T-cells and an upregulation of PD-1 in CD8 **+** T-cells, contributing directly and indirectly to immune evasion [130].

### Macrophages undergo phenotypic shifts during metabolic rewiring in GBM

**Fig. 2:**
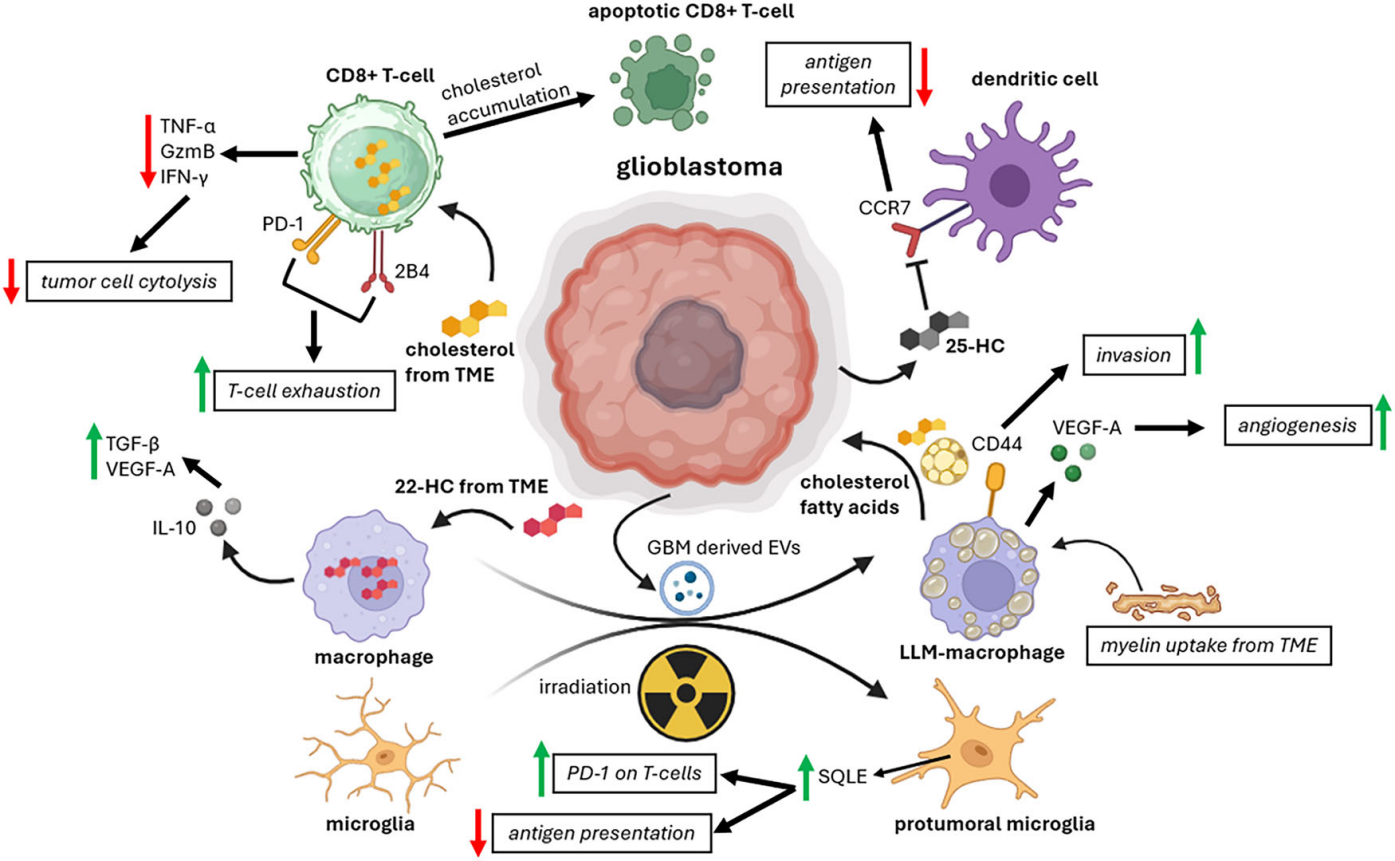
Accumulation of cholesterol in CD8 + T-cells leads to T-cell exhaustion and apoptosis, thereby hampering T-cell-mediated antitumor immunity. Secretion by tumor cells of the cholesterol metabolite 25-HC impairs antigen presentation through inhibition of CCR7 on dendritic cells. Uptake of 22-HC from the TME by macrophages promotes M2 macrophage polarization. LLM macrophages are a subset of macrophages that display increased myelin uptake from the TME and degradation thereof, enabling them in providing cholesterol and fatty acids to GBM cells. Epigenetic changes in these LLMs lead to an increased expression of anti-inflammatory genes like ATF3 and C/EBPβ, inhibiting antitumor immune responses. GBM-derived extracellular vesicles promote lipid droplet accumulation in glioma-associated macrophages, leading to decreased phagocytic activity, CD44 upregulation and angiogenesis. Oxidative stress promotes SQLE expression in GBM-associated microglia, leading to an increase in PD-1 expression on CD8 + T-cells and decreased antigen presentation. ATF3: activating transcription factor 3, C/EBPβ: CCAAT enhancer binding protein beta, 25/27-HC: 25/27-hydroxycholesterol, IL-10: interleukin 10, TGF-β: transforming growth factor beta, VEGF: vascular endothelial growth factor, CCR7: C-C motif chemokine receptor 7, PD-1: programmed cell death 1, 2B4: CD244 molecule, TNF-α: tumor necrosis factor alpha, GzmB: granzyme B, IFN-γ: interferon gamma. CD44: hyaluronate receptor, SQLE: squalene epoxidase. Figure created with BioRender.com.

Tumor-associated macrophages (TAMs) can consist up to 30-50% of the total mass in GBM, and it comes as no surprise that metabolic rewiring in this myeloid cell subset plays a pivotal role in tumor growth and immune-evasion [131, 132]. Treatment of macrophages with the cholesterol metabolite 27-hydroxycholesterol (27-HC) led to the release of the anti-inflammatory cytokine IL-10 and polarized macrophages toward the tumor-supporting M2 phenotype [133]. More recently, a study by Kloosterman et al. revealed that a distinct subtype of macrophages plays multiple roles in supporting GBM growth and proliferation. In the hypoxic niche of recurrent post-irradiated GBM, an increased infiltration of lipid-laden macrophages (LLMs) was observed in vivo. These LLMs exhibit a high metabolic turnover of myelin, providing GBM cells with fatty acids and cholesterol. Furthermore, they display elevated expression of transcription factors involved in immunosuppression (i.e., ATF3 and C/EBPβ) [134]. Governa et al. corroborated these findings in another context by demonstrating that macrophages exposed to GBM-derived extracellular vesicles (EVs) obtain a lipid-laden phenotype and upregulate CD44 and VEGF-A, promoting GBM invasion and angiogenesis, respectively [135]. Another study emphasizing the role of cholesterol in immune evasion shows that the generation of 25-hydroxycholesterol (25-HC) inhibits antitumor activity of macrophages by skewing their polarization toward the M2 phenotype through STAT6 activation [136]. These observations advocate for a paradigm shift from targeting solely tumor cells to also targeting macrophage subsets that support GBM in multiple ways. In summary, fatty acids and cholesterol can contribute to the tumor’s ability to create an immune-evasive environment by inhibiting CD8 **+** T-cell function and promoting the polarization of myeloid cells to a tumor-supporting phenotype (Fig. 2).

## Fatty acid metabolism in GBM cells is a complex interplay

Cholesterol and fatty acids are not directly synthesized from one another as they are produced through distinct metabolic pathways. However, their metabolic activity can influence each other strongly through interconnected pathways. Therefore, discussing fatty acids alongside cholesterol is essential to uncover broader strategies in the quest for novel targets to combat GBM.

### GBM cells undergo a lipogenic switch

Fatty acids (FA) play a crucial role in cellular homeostasis and maintenance of proper protein function. It has been established that de novo lipogenesis (coined the lipogenic switch) allows cancer cells to sustain rapid proliferation and to meet the need for more autonomy [137, 138]. In the CNS, normal human astrocytes (NHA) rely mostly on exogenous lipids to maintain their vital functions in brain physiology. These exogenous lipids are derived from the circulation and are able to cross the BBB via the action of fatty acid transporters as FATP1, FATP4, and CD36 [139–141]. GBM cells have been observed to rewire their metabolic pathways to favor de novo lipogenesis, indicating that the fatty acid supply provided by the environment may not be sufficient to maintain its metabolic needs [142].

### Rewired fatty acid metabolism modulates apoptosis sensitivity and antitumor immunity

Glioblastoma is known to evade apoptosis through multiple ways. These include, but are not limited to, overexpression of anti-apoptotic proteins, downregulation of pro-apoptotic proteins and mutations in p53 [143, 144]. Emerging findings imply a protumoral role of lipid metabolism rewiring in GBM, albeit context and lipid species-dependent. Cortez et al. demonstrated that upregulation of fatty acid binding protein 7 (FABP7) protects GBM cells from ferroptosis but promotes apoptosis in CD8 **+**T-cells, hampering the infiltration of the latter and hereby exerting bidirectional roles dependent on the cell type [145]. Stearoyl-CoA desaturase (SCD1), a protein involved in the generation of mono unsaturated fatty acids, was found to be essential in protecting GBM cells from ER stress-induced apoptosis and maintenance of stemness through expression of GSC markers SOX2 and nestin [146]. Furthermore, diacylglycerol O-acyltransferase 1 (DGAT1) inhibition has been demonstrated to promote cytochrome c release and subsequent apoptosis in GBM cells, indicating a protective effect of DGAT1 to apoptosis in GBM [147]. Rewired lipid metabolism might promote immune evasion in GBM as well, although the evidence hereof remains scarce. TNF receptor-associated factor 3 (an inducer of lipid peroxidation, TRAF3) downregulation was demonstrated to sensitize GBM cells to PD-L1 inhibition in vivo [148]. Altogether, the current evidence of lipid metabolism rewiring in GBM hints toward a protumoral role in GBM, and targeting this rewiring might exert antitumor effects.

### FASN is a key player in GBM growth, stemness, and angiogenesis

The key players involved in the rewired fatty acid metabolism in glioblastoma (GBM) remained unclear for a long time. FASN, an enzyme located in the cytosol of mammalian cells, plays a crucial role in de novo fatty acid synthesis, a process frequently upregulated in cancer cells [149]. FASN expression was found to correlate with the WHO grade of gliomas, prompting further investigation into its role in GBM [150]. FASN appears to be regulated by several upstream activators, including EGFR and therapy-induced energetic stress [151, 152]. Particularly irradiation has been shown to promote FASN activity^194^. FASN activity was also revealed to be a consequence of ubiquitin-specific peptidase 2 (USP2a) overexpression in GBM relative to normal tissue, leading to inhibition of (ubiquitinated) FASN degradation [153]. Zhao et al. found that cerulenin (targeting FASN) reduced glioma cell viability in vitro, accompanied by an S-phase arrest and increased levels of cleaved PARP. These effects were specific to GBM cells, with no impact on astrocytes [154]. Inhibition of FASN was also shown to downregulate S-phase kinase-associated protein 2 (SKP2), an essential mediator of p27 proteasomal degradation [155]. This suggests that FASN also promotes cell cycle progression in GBM cells.

**Fig. 3:**
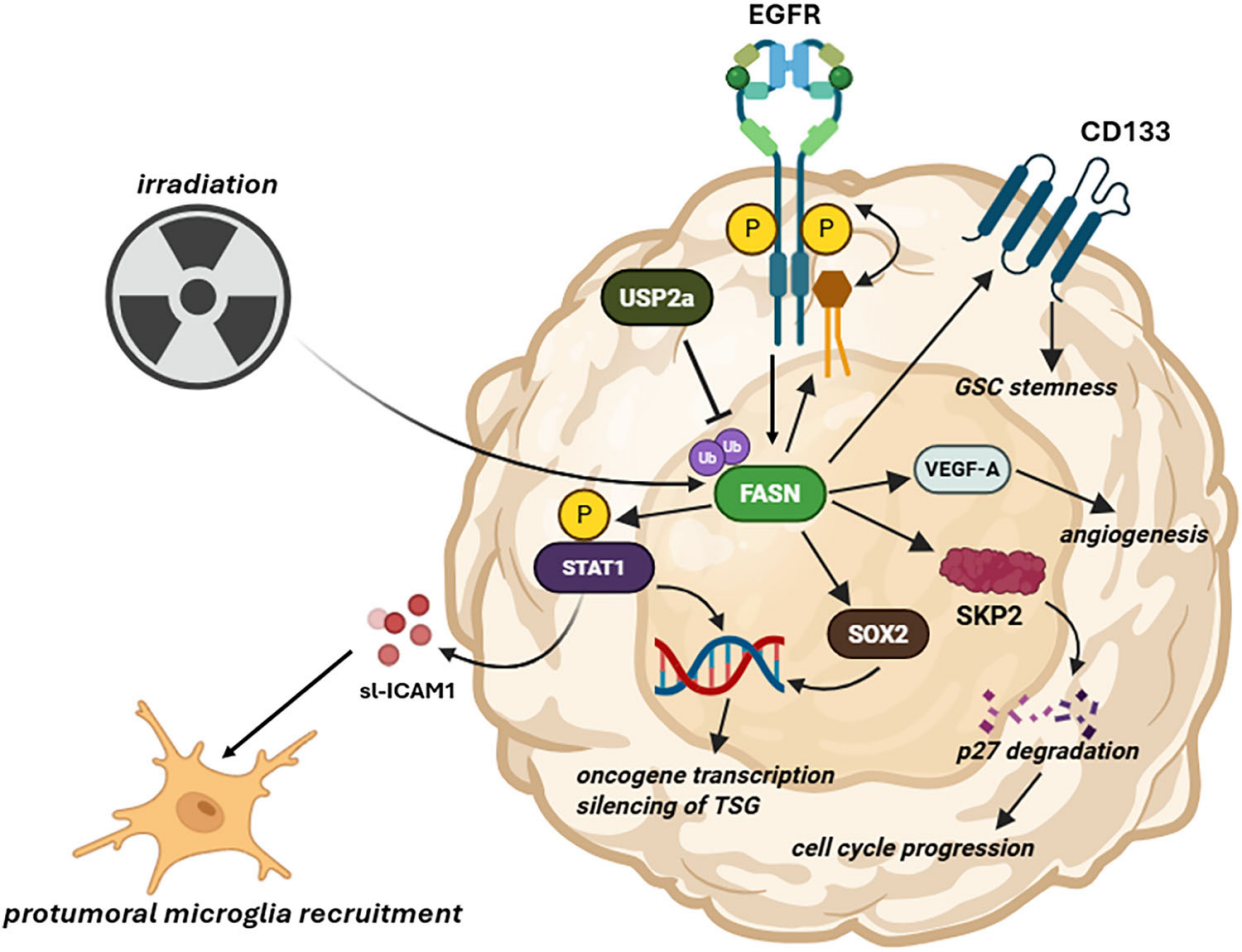
FASN mediates a wide variety of oncogenic signaling events in GBM. Irradiation, hyperactivity (be it ligand-dependent or independent) of EGFR and USP2a expression stimulate FASN activity. For simplicity, only three upstream activators are shown for FASN, while cholesterol and fatty acid depletion are also able to induce FASN activity. USP2a inhibits FASN degradation and leads to sustained FASN activity. Upregulation of FASN promotes EGFR activity (through palmitoylation of its intracellular tail), cell cycle progression, angiogenesis, and stemness. FASN upregulation promotes phosphorylation of STAT1, leading to its nuclear translocation and transcription of genes that promote soluble ICAM1 expression. Secretion of ICAM1 promotes recruitment of glioblastoma-associated microglia. GSC glioma stem-like cells, TSG tumor suppressor genes, EGFR epidermal growth factor receptor, FASN fatty acid synthase, CD133 prominin-1, VEGF-A vascular endothelial growth factor alpha, p27 cyclin-dependent kinase inhibitor 1B, SKP2 S-phase kinase-associated protein 2, SOX2 SRY-box transcription factor 2. STAT1 signal transducer and activator of transcription 1, sl-ICAM1 soluble ICAM1, USP2a ubiquitin-specific peptidase 2a. Figure created with BioRender.com.

FASN might also promote stem-like behavior of GBM cells. Yasumoto et al. demonstrated that inhibition of FASN led to a substantial decrease in glioma stem cell markers SOX2, nestin, and CD133 [156]. This finding was reproduced for SOX2 in vitro and in vivo in a neuroblastoma model [157]. Neural stem and progenitor cells (NSPCs) require FASN to maintain their self-renewing capacity and proliferation [158]. Thus, FASN might be indispensable for both GBM cells and NSPCs to maintain their stemness and proliferative potential [159, 160]. Moreover, Zhou et al. demonstrated that FASN inhibition decreased the expression of angiogenesis inducers HIF-1α and VEGF-A in vivo, possibly via upregulation of an anti-angiogenic isoform of VEGF-A (VEGF165b) [161, 162]. The observation that FASN has a myriad of functions in GBM cells is further substantiated by the finding that EGFR constitutive activity can be achieved by palmitoylation (covalent attachment of a palmitic acid group) of tyrosine residues on EGFR intracellularly, a process possibly promoted by FASN [163]. Although current evidence is scarce, FASN activity might also influence the interaction between GBM and the TME. Zhang et al. found that FASN activity promoted STAT1 phosphorylation and recruitment of protumoral microglia [164]. The above-described phenomena all hint toward a tumor-promoting role of FASN (Fig. 3).

### Peroxisome proliferator-activated receptors in GBM: a double-edged sword

Peroxisome proliferator-activated receptors (PPARs) are nuclear receptors that mediate fatty acid uptake, oxidation, and binding of DNA elements to modulate a wide variety of signaling events [165]. The most studied variants of these nuclear receptors are PPARα and PPARγ, as they influence many lipogenic signal transduction pathways. PPARα is characterized by its stimulatory effect on fatty acid oxidation in mitochondria, peroxisomes, and microsomes while also mediating fatty acid uptake and lipoprotein synthesis. PPARγ is involved in the catabolism of fatty acids, inhibition of cholesterol synthesis, and modulation of inflammatory mediators [166–168]. Although the extent to which PPARs support GBM growth and proliferation remains unclear, emerging research highlights their potential to either promote or inhibit GBM progression.

Haynes and Benedetti et al. demonstrated that PPARα is overexpressed in GBM tissue and correlated with GBM grade [169, 170]. Especially the hypoxic niche of glioma shows a higher expression of PPARα, indicating that hypoxia might promote PPARα activity and transcription of its target genes [171]. One of the target genes of PPARα is carnitine palmitoyl transferase 1C (CPT1C), which is involved in metabolic reprogramming of tumor cells [172]. Another target gene of PPARα is ubiquitin-like with PHD and ring finger domains 1 (UHRF1) [173]. Various studies have demonstrated that UHRF1 plays a role in epigenetic silencing of tumor suppressor genes (TSG), further supporting a tumor-promoting role of PPARα [174, 175]. Importantly, PPARα expression is able to promote fatty acid oxidation (FAO) [176, 177]. FAO, in turn, is implicated in many oncogenic signaling events, where the most common ones will not be described in this review [178, 179]. In terms of immune evasion, FAO has been demonstrated to promote CD47 expression, which impairs the phagocytic activity of macrophages by functioning as a ‘don’t eat me’ signal [180]. The observation that FAO promotes M2 macrophage polarization and inhibition of T-cell proliferation further supports the notion that FAO promotes immune evasion [181, 182]. These immune evasion tactics could thus be promoted by PPARα activity. Finally, Fidoamore et al. demonstrated that PPARα antagonism decreased the formation of GBM neurospheres and lipid droplet content in GBM cells [183].

**Fig. 4:**
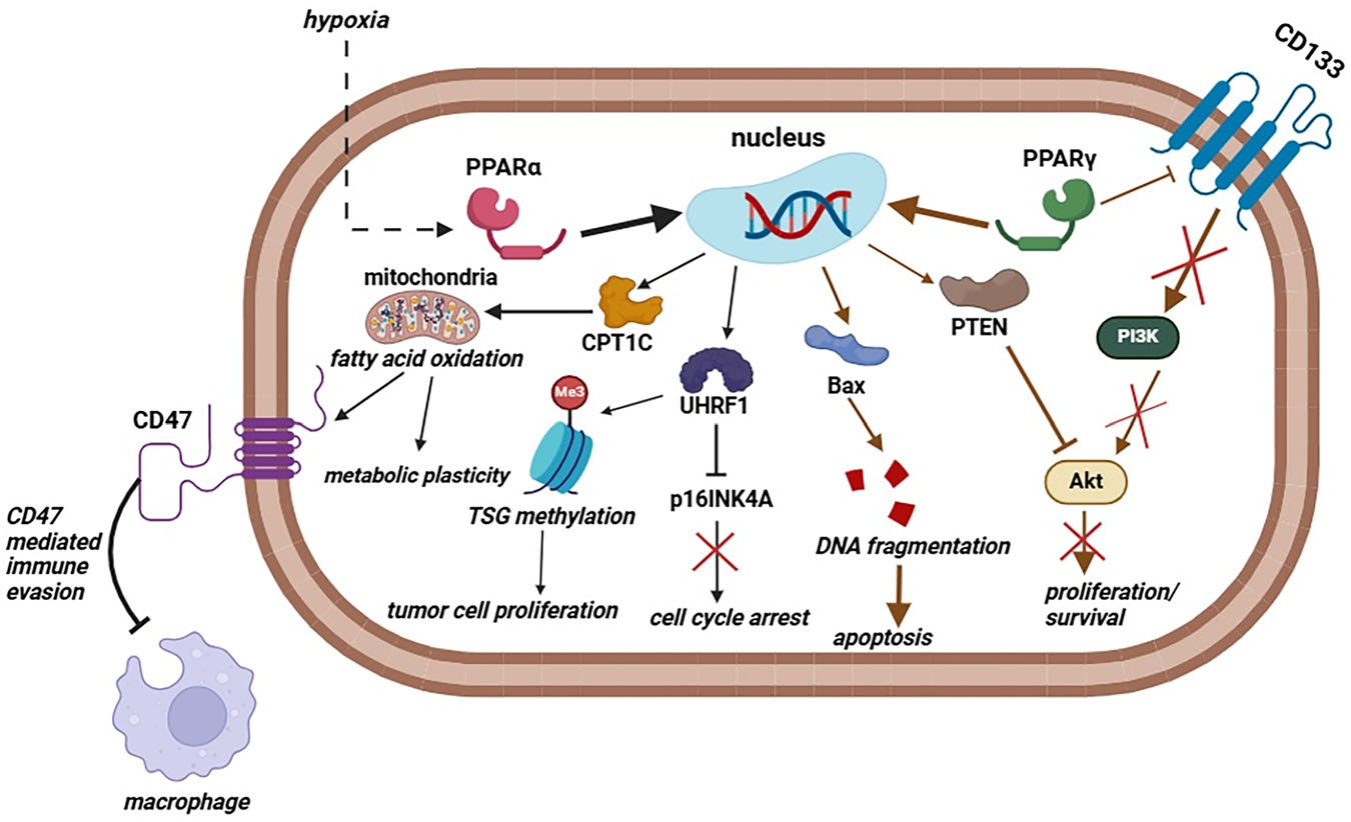
Proposed roles of PPARα and PPARγ in GBM. In the case of PPARα, stimuli like hypoxia can trigger its nuclear translocation for activation of its target genes (black arrows). This activation of target genes leads to growth and proliferation of GBM cells through various mechanisms. Upregulation of CD47 is also able to increase UHRF1 expression (not shown in this figure for clarity). PPARγ activity leads to downregulation of CD133, inhibition of the PI3K/Akt pathway, and induction of apoptosis (blue arrows). Bax BCL2-associated X, apoptosis regulator, CPT1C carnitine palmitoyltransferase 1C, UHRF1 ubiquitin-like with PHD and ring finger domains 1, PI3K phosphoinositide-3-kinase, PTEN phosphatase and tensin homolog, CD133:prominin-1, p16INK4A cyclin-dependent kinase Inhibitor 2A, TSG tumor suppressor genes. Figure created with BioRender.com.

PPARγ, in contrast to PPARα, exerts antitumoral effects in GBM cells. Chearwae et al. demonstrated that upon administration of the PPARγ agonist 15d-PGJ2, GBM cells showed impaired sphere formation, CD133 downregulation, lower EGFR activity, and induction of apoptosis [184]. Bridging old players and new players, Patel et al. revealed that PPARγ is able to induce the transcriptional activity of the gene encoding PTEN, which is still intact in a subgroup of GBM patients [185, 186]. PPARγ has also been shown to promote apoptotic signaling through downregulation of Akt activity and upregulation of Bax and Bad proteins in GBM cells [187, 188]. However, in an immunological context, PPARγ activation leads to macrophage polarization towards the M2 phenotype, implying a role of this PPAR in immune evasion [189, 190]. Despite belonging to the same family of nuclear hormone receptors, there are strong implications that PPARs have a role in the inhibition (PPARγ) or promotion (PPARα) of glioma cell growth and proliferation, depending on the subtype of this nuclear receptor and the cell type in which the PPAR is active (Fig. 4).

### Cholesterol and lipid metabolism rewiring contribute to radiochemoresistance

Sensitivity to radiochemotherapy can be affected by GBM metabolic rewiring to render tumor cells less sensitive to cytotoxic treatments. De Martino et al. demonstrated that irradiated GBM cells promote the accumulation of unsaturated fatty acids in lipid droplets, thereby protecting tumor cells from endoplasmic reticulum (ER) stress [191]. Another study revealed that TMZ-resistant GBM cells display an upregulation of specificity protein 1 (SP1), the latter promoting the conversion of arachidonic acid to prostaglandin E2 (PGE2). PGE2 activity in turn was demonstrated to induce FAO and altered mitochondrial dynamics, most likely to increase the threshold necessary for apoptosis induction upon DNA damage [192, 193]. These findings emphasize the complexity of radiochemoresistance by indicating that DNA repair-independent pathways are also at play to mitigate therapeutic effects in GBM. In addition, in vitro targeting of tyrosine kinases with ponatinib led to an upregulation of LDLR and an increase in enzymes necessary for de novo cholesterol synthesis, further strengthening the proposed involvement of cholesterol and lipid metabolism (through enhanced uptake and de novo synthesis) in counteracting the cytotoxic effects of drugs, irrespective of their mechanism of action [194, 195]. Altogether, rewiring of cholesterol and lipid metabolism in GBM can contribute to the development of therapeutic resistance and offers a target to sensitize GBM to therapy.

## Repurposing drugs that target cholesterol and lipid metabolism

While no current chemotherapeutic/anticancer agent specifically targets cholesterol or lipid metabolism, several approved non-oncological drugs targeting these pathways are being explored in cancer research. Among these, statins are the most extensively studied, demonstrating antitumor effects in multiple types of cancer through mechanisms of apoptosis induction, cell cycle arrest, and autophagy [196–198]. Mohapatra et al. showed that fluvastatin potentiated radiation-induced cell death by modulating the DNA repair capacity in pancreatic cancer cells [199]. Similarly, pitavastatin sensitized melanoma and breast cancer cells to radiation by impairing DNA double-strand break repair in vivo, suggesting off-target effects unrelated to HMGCR inhibition [200]. Importantly, pitavastatin exerted cytotoxic and synergistic effects with temozolomide in an in vitro model of GBM [201]. The mentioned observations here are a proportion of all the studies demonstrating antitumor effects of statins, and discussing all of them is beyond the scope of this review article.

Aside from statins, the FASN inhibitor orlistat revealed antitumor effects including apoptosis induction, PD-L1 downregulation, and inhibition of STAT3-NF-kB signaling [202–204]. Ezetimibe, a drug that inhibits cholesterol uptake in the intestine, has exhibited antitumor effects in vitro through mechanisms distinct from other cholesterol-targeting drugs [205–207]. Table 1 highlights some of the key non-oncological drugs that target cholesterol and/or lipid metabolism and have been studied for their potential antitumor effects. The table lists the lowest cytotoxic concentrations and the maximum plasma concentrations observed in patients. The ratio between these values indicates the potential feasibility of achieving therapeutic effects, as cytotoxic levels exceeding plasma levels are often unachievable without toxic side effects. However, for GBM, drugs must also possess favorable physicochemical properties that allow penetration of the BBB. The CNS-multiparameter optimization score (CNS-MPO) is an indicator of a drug’s likeliness to cross the BBB (the closer to 6.0 the better) [208].

**Table 1:** An overview of non-oncological drugs targeting cholesterol and lipid metabolism.

Drug	Current indication	Validated target	Antitumor concentration in vitro* (µM) [ref]	Tumor type	Plasma concentration (µM)** [ref]	Ratio [antitumor]/[plasma]	CNS-MPO score	GBM trial? [ref]
Atorvastatin	Hypercholesterolemia	HMGCR	10 [197]	glioblastoma	0.0065 [213]	1538	2.1	Yes [208]
Fluvastatin	Hypercholesterolemia	HMGCR	9.0 [199]	pancreatic	0.8978 [214]	10	4	No
Lovastatin	Hypercholesterolemia	HMGCR	5.0 [215]	glioblastoma	0.0099 [216]	505	4	No
Pitavastatin	Hypercholesterolemia	HMGCR	0.66 [201]	glioblastoma	0.1591 [217]	4.1	3.9	Yes [211]
Pravastatin	Hypercholesterolemia	HMGCR	50 [218]	hepatocellular	0.15 [219]	333	3.5	No
Rosuvastatin	Hypercholesterolemia	HMGCR	14 [220]	prostate	0.0394 [221]	355	3.3	No
Simvastatin	Hypercholesterolemia	HMGCR	4.0 [222]	glioblastoma	0.0406 [223]	99	3.6	No
Orlistat	Obesity	FASN	18 [162]	breast	0.303 [224]	59	2.9	No
Ezetimibe	Hypercholesterolemia	NPC1L1, SOAT1 and ANPEP	38 [205]	breast	0.054 [225]	704	3.2	No
Fenofibrate	Hyperlipidemia	PPARα	25 [226]	glioblastoma	26 [227]	0.96	4	No
Pioglitazone	Diabetes mellitus type 2	PPAR-γ	30 [187]	glioblastoma	2.61 [228]	11.4	5.3	Yes [212]

*lowest cytotoxic concentration found in vitro, **highest observed plasma concentration after administration of prescribed doses for the management of the indication displayed. *CNS-MPO* central nervous system multiparameter optimization score, *HMGCR* HMG-CoA reductase, *FASN* fatty acid synthase, *LIPF* Lipase F, gastric type, *PNLIP* pancreatic lipase, *SOAT1* sterol O-acyltransferase 1, *ANPEP* alanyl (membrane) aminopeptidase.

Some drugs targeting cholesterol and lipid metabolism have advanced to GBM clinical trials, reflecting ongoing efforts to explore their efficacy in targeting cholesterol metabolism in GBM (Table 2). Two statins, atorvastatin (ATV) and lovastatin (LVS), did not improve OS in either monotherapy or combination therapy. Notably, a non-significant increase in 1-year survival to 75% was observed in the ATV plus TMZ group, compared to 64% in the control group [209]. A new trial investigating the efficacy of ATV (20 mg orally) is underway (NCT06327451). Lovastatin, although showing a minor signal of efficacy (four patients achieved disease stabilization) did not improve OS [210]. Results from the phase 0 trial of pitavastatin (NCT05977738), have not been published yet [211]. Pioglitazone in combination with rofecoxib (COX-2 inhibitor) and TMZ did not improve OS, although two patients achieved a partial response lasting 32-44 months [212]. This study included fourteen patients, of whom four had anaplastic astrocytoma and ten had GBM, which may have influenced the observed results. In summary, future clinical trials are of great importance to gain insight into the clinical activity of approved non-oncological drugs that target cholesterol and lipid metabolism.

**Table 2:** A summary of clinical trials aimed at investigating the clinical efficacy of drugs targeting cholesterol and lipid metabolism in GBM.

Drug	Trial design	Inclusion	Safety	Results	Conclusion
Pioglitazone	Phase I-II: capecitabine + rofecoxib + pioglitazone **or** TMZ + rofecoxib + pioglitazone	Recurrent High-grade astrocytoma (*n* = 14), primary GBM (*n* = 10) and AA (*n* = 4)	Grade 3 events (*n* = 2)	PR *n* = 2. Duration PR > 32 months	Terminated due to lack of efficacy
Atorvastatin	Phase II: ATV + TMZ + RTx. After RTx continuation with TMZ alone **or** TMZ + ATV	Newly diagnosed GBM patients (*n* = 36)	Grade 3 events (*n* = 7)	SD *n* = 7	No significant improvement in OS
Atorvastatin	Phase II: ATV + RTx + TMZ **or** RTx + TMZ (Stupp)	Newly diagnosed GBM (*n* = 50)	NA	NA	Recruiting
Lovastatin	Phase I-II: 1 week on and 3 weeks off (recurrent GBM) **or** combined with Stupp (primary GBM)	Recurrent and primary GBM	Grade 2 events (*n* = 2)	PR = 1, MR = 1 and SD = 1	No significant improvement in OS
Pitavastatin	Phase 0: 16, 32, or 48 mg PVT a day for 6 days pre-operatively	Newly diagnosed and recurrent GBM	NA	NA	Completed, results not published yet

*AA* anaplastic astrocytoma, *MR* minor response, *SD* stable disease, *PR* partial response, *ATV* atorvastatin, *AA* anaplastic astrocytoma, *OS* overall survival, *PVT* pitavastatin, *NA* not applicable.

## Conclusion and future perspectives

Glioblastoma remains one of the most challenging cancers to combat with survival rates stagnating since the approval of the current standard of care in 1999. In this review, we sought to shed light on the intricacies of cholesterol and lipid metabolism by not only considering intracellular signaling pathways modulated by this rewired activity but also describing its broader impact on the TME. We further provided an overview of approved non-oncological drugs that modulate cholesterol and lipid metabolism and which have been investigated for their potential antitumor effects in GBM. Translating the observed in vitro effects, particularly those of statins, into therapeutic benefits for GBM patients requires validation through clinical trials. Not all drugs possess CNS-MPO scores indicative of adequate BBB penetration. For those that do, standard dosing regimens used for cholesterol/lipid management may be insufficient to achieve the concentrations necessary for direct antitumor effects in GBM. Additionally, cytotoxic effects observed in in vitro studies are typically assessed after a single or short-term treatment, whereas in patients, these drugs are dosed daily, and clinical effects may emerge more slowly. In this context, combination studies with irradiation and/or TMZ remain crucial in evaluating the potential of these drugs as add-ons to the standard of care. Finally, future drug discovery efforts in GBM should aim to develop new or repurposed drugs that not only target these dysregulated metabolic signaling pathways but also possess physicochemical properties that enable BBB penetration.
